# The Effects of Mindfulness on Shame: Exploring Mediation by Cognitive Flexibility and Self‐Compassion in a Chinese Adult Population

**DOI:** 10.1002/pchj.817

**Published:** 2024-12-10

**Authors:** Xiaoshuo Zhang, Jinghong Wang, Yuzheng Wang, Jinyan Wang, Fei Luo

**Affiliations:** ^1^ CAS Key Laboratory of Mental Health Institute of Psychology Beijing People's Republic of China; ^2^ Department of Psychology University of Chinese Academy of Sciences Beijing People's Republic of China

**Keywords:** cognitive flexibility, mindfulness, self‐compassion, shame

## Abstract

To examine the effects of mindfulness on shame and the mechanisms mediated by cognitive flexibility and self‐compassion in a Chinese adult population in daily life, we conducted two studies. Study 1 was a cross‐sectional study using the Five‐Factor Mindfulness Questionnaire, the Cognitive Flexibility Inventory, the Self‐Compassion Scale, and the Self‐Conscious Affect‐3, which were administered to 481 adults in Beijing and Chengdu. For Study 2, an 8‐month follow‐up study was conducted on 128 of the adults. The results of Study 1 showed that (1) the awareness of action and nonjudgment dimensions, and the total score of mindfulness were significantly correlated with shame; (2) cognitive flexibility and self‐compassion could fully mediate the prediction of mindfulness on shame. The Study 2 showed that (1) mindfulness and shame were significantly negatively correlated in both phases of measurement; (2) controlling for T1 shame, T1 mindfulness was able to negatively predict T2 shame; controlling for T1 mindfulness, T1 shame was not able to predict T2 mindfulness. There is a longitudinal association between mindfulness and shame, and only mindfulness scores are predictive of the shame and not vice‐versa; both cognitive flexibility and self‐compassion can provide explanations for the prediction of shame by mindfulness. Enhancing levels of mindfulness can help alleviate individuals' shame levels.

## 1 Introduction

### 1.1 Shame and Mindfulness

With the increasing development of digitalization and the Internet, various social media and short video platforms have become indispensable social tools in people's lives, and have made the interaction between individuals and society closer (China Internet Network Information Center 2024). Some scholars have suggested that shame is related to excessive use of social media (Fioravanti et al. 2023; Gioia, Griffiths, and Boursier 2020). In modern society, where the criteria for evaluating individuals have become increasingly diverse, individuals may experience shame when they feel they have not met these standards (Lewis 1971). Social media platforms provide a stage for self‐presentation and evaluation of others, and this interactive model increases the opportunities for individuals to be judged, thereby raising the risk of not meeting these evaluative criteria and potentially exacerbating the experience of shame. For example, since esthetic standards are often not unified, an individual may experience body shame if they internalize these unattainable standards as measures of their own worth (Kim, Seo, and Baek 2014). Therefore, while social media provides immediate and abundant information resources, it also exposes people to the possibility of shame at any time.

Shame is a self‐conscious emotion (SCE), which arises when an individual develops an awareness of the self and is generated through self‐awareness, self‐evaluation, and self‐reflection (Lewis 1995). According to the attribution theory of emotions, SCE occurs when individuals attribute the cause of a triggering event to something within themselves (Lewis, Haviland‐Jones, and Barrett 2008; Tangney and Dearing 2002; Weiner 1985). Shame occurs when the event is inconsistent with the individual's goals and the focus of the internal attribution is a negative evaluation of the “self,” and guilt occurs when the focus of the internal attribution is a negative evaluation of the behavior the self has done (Lewis 1971). Although both shame and guilt are emotional states that individuals do not want to experience or even loathe, they differ in both emotional experience and behavioral tendencies (Tangney and Dearing 2002). Shame is more likely to lead to distress and a range of psychological problems. In recent years, a number of systematic reviews and meta‐analyses have confirmed that compared to guilt, shame is associated with anxiety (Candea and Szentagotai‐Tata 2018), depression (Kim, Thibodeau, and Jorgensen 2011), self‐mutilation/self‐harm (Sheehy et al. 2019), social anxiety (Swee, Hudson, and Heimberg 2021), etc. Some data also suggest that shame is positively associated with alcohol and drug abusing problems (Dearing, Stuewig, and Tangney 2005), and eating disorders (Kelly and Carter 2013). Managing and intervening in shame is evidently crucial for everyone in modern society. Therefore, this study focuses on and explores shame and its interventions.

Mindfulness is the purposeful, nonjudgmental awareness of the here and now experience (Kabat‐Zinn 2003), which emphasizes awareness of the current experience and promotes a nonjudgmental attitude that allows all experiences to exist naturally. Mindfulness has been repeatedly validated for its benefits in improving emotion regulation (González‐Martín et al. 2023; Hofmann et al. 2010) and improving physical and mental health (Keng, Smoski, and Robins 2011; Liu et al. 2022). Mindfulness can also reduce negative emotions. A meta‐analysis indicates that compared to control groups, mindfulness interventions can better improve anxiety and depression emotions (Shi et al. 2023). A systematic review of shame interventions describes the major approaches available to intervene with shame, which include mindfulness, cognitive behavioral therapy, and self‐compassion therapy (Goffnett, Liechty, and Kidder 2020). The utility of mindfulness for reducing shame has been demonstrated in various groups: 8 weeks of mindfulness cognitive therapy reduced shame tendencies in anxious and depressed patients (Proeve, Anton, and Kenny 2018); infertility patients who underwent 10 weeks of mindfulness training also had reduced shame (Galhardo, Cunha, and Pinto‐Gouveia 2013); and mindfulness stress‐reduction therapy was effective at reducing shame in posttraumatic stress disorder patients (Goldsmith et al. 2014). A 9‐week mindfulness‐based trauma recovery for refugees reduced shame in trauma recovery among African asylum‐seekers (Oren‐Schwartz et al. 2022). All of these studies exemplify the effectiveness of mindfulness as an intervention for shame. However, these studies were mostly conducted in Western countries and were targeted at heterogeneous populations.

Although the mitigating effects of different interventions based on mindfulness on shame have been supported by the results of many studies, the mechanisms involved in the onset of the effects are currently inconclusive. Lindsay and Creswell (2017) proposed that mindfulness training would help individuals engage in the direct experience of internal and external feelings in the present moment without getting caught up in overthinking by focusing attention on the awareness of moment‐to‐moment experiences in a nonjudgmental manner (Teasdale, Segal, and Williams 1995). This approach is diametrically opposed to the shame‐driven avoidance of current experience and a comprehensive and negative evaluation of the self (Tangney, Wagner, and Gramzow 1992). Therefore, it is hypothesized that such a process can reduce the rejection of current experiences and the denial of the self in shame‐prone individuals, thereby alleviating shame levels.

Hypothesis 1 *Mindfulness negatively predicts shame*.

### 1.2 The Mediating Role of Cognitive Flexibility and Self‐Compassion

Cognitive flexibility is an individual's ability to respond flexibly and adaptively in the face of different stimuli or changes in the environment (Dennis and Vander Wal 2010). A study with a group of college students showed that higher cognitive flexibility was associated with lower shame after experiencing a potentially morally harmful event (Cenkner, Usman, and Zalta 2023). Moreover, lower levels of cognitive flexibility significantly increased the likelihood of belonging to the shame prominent profile (Cenkner, Held, and Zalta 2024). Mindfulness has been found to be significantly and positively correlated with cognitive flexibility (Moore and Malinowski 2009). A neuroscientific systematic review of mindfulness interventions has preliminarily confirmed that mindfulness training could enhance cognitive flexibility (Lao, Kissane, and Meadows 2016). Bishop et al. (2004) suggested that mindfulness is operationally defined as the self‐regulation of attention and orientation to the experience. Being cognitive flexible is considered an important component of self‐regulation of attention (Zou et al. 2020). Some studies have further suggested that the improvement of cognitive flexibility is an important mechanism through which mindfulness alleviates negative emotions. For example, Shapero et al. (2018) found that mindfulness training can alleviate depression by enhancing cognitive flexibility in patients with depression. Yousefi Afrashteh and Hasani (2022) found that cognitive flexibility played a mediating role in the effect of mindfulness on the mental health in adolescents. Wen et al. (2021) indicated that cognitive flexibility mediated the effect of mindfulness on stress and anxiety in elementary school students. Therefore, mindfulness could enable individuals to shift cognitive sets more flexibly in response to the changing external and internal stimuli, resulting in less negative emotional reactivity or more adaptive emotion regulation (Moore and Malinowski 2009; Wen et al. 2021). Thus, we predict that the level of cognitive flexibility plays a key role in the predictive effect of mindfulness on shame. That is, individuals with high levels of mindfulness possess higher cognitive flexibility and have lower levels of shame.

Hypothesis 2 *Cognitive flexibility mediates the relationship between mindfulness and shame*.

According to Bishop et al. (2004), mindfulness is the process of attending to the present moment and the self with openness and acceptance, and this process is an adaptive behavior. Self‐compassion is the ability to hold adaptive attention to the self in difficult situations (Neff 2003a). Mindfulness and self‐compassion both emphasize the awareness of present experiences and approach both oneself and these experiences with a nonjudgmental attitude. However, their core focuses are different. The core focus of mindfulness is on the awareness and acceptance of present experiences, which may be good, bad, or neutral. Self‐compassion, on the other hand, is more about being aware of one's own needs when facing suffering and providing oneself with warmth and support (Neff and Germer 2013). Regardless of the situation, the prerequisite for self‐compassion is that an individual can recognize they are in pain (Neff 2023). In other words, mindfulness is the foundation that allows an individual to alleviate shame through self‐compassion. Some research has confirmed that Mindfulness Based Stress Reduction Therapy can enhance an individual's level of self‐compassion (Barnard and Curry 2011). According to Neff's (2003b) definition, self‐compassion consists of three components: self‐kindness, the sense of common humanity, and mindfulness. Mosewich et al. (2011) argued that these three dimensions contrasted with the trait of shame: (1) shame‐prone individuals often focus on their own flaws and mistakes (Lewis 1995) and rarely express kindness to the self; (2) they are overly focused on the self that experiences failures (Lewis 1995), while the sense of common humanity emphasizes the fact that everybody will fail or make mistakes; (3) they will overly deny themselves because of their failures (Tangney and Dearing 2002), and mindfulness in self‐compassion emphasizes not overidentifying with the experience of the present moment. Therefore, in addition to mindfulness intervention, self‐compassion is also an important way to regulate shame (Gilbert 2009; Goffnett, Liechty, and Kidder 2020; Westerman, McCann, and Sparkes 2020). Kelly and Waring (2018) reported a reduction in shame levels in anorexia nervosa patients after 2 weeks of self‐compassionate letter‐writing. Highly shamed college students also experienced a significant reduction in shame after 16 days of self‐compassionate letter‐writing (Swee et al. 2023). In the general nonclinical patient population, Fink‐Lamotte et al. (2023) also confirmed that self‐compassion exercises are an important intervention for reducing shame. Previous studies have found that self‐compassion mediates the effect of mindfulness on shame (Sedighimornani 2019; Woods and Proeve 2014). That is, mindfulness decreases shame by enhancing individuals' self‐compassion. Therefore, we hypothesize that self‐compassion plays a key role in the effects of mindfulness on shame.

Hypothesis 3 *Self‐compassion mediates the relationship between mindfulness and shame*.

In addition to this, some studies have also found a relationship between cognitive flexibility and self‐compassion (Martin, Staggers, and Anderson 2011; Okan Er and Deni̇Z 2022; Zeidan et al. 2015). Neff and McGehee (2010) had a research on college students, and it has found that family and cognitive factors were identified as predictors of individual differences in self‐compassion. That is, college students who can recall warm and supportive caregivers tend to have higher levels of self‐compassion. We believe that individuals with high cognitive flexibility have more cognitive resources and are more likely to retrieve these supportive memories when facing difficulties, thus they are more likely to have higher levels of self‐compassion. If an individual has low cognitive flexibility, even if they have such supportive memories, they may not be able to access them in times of distress to help themselves, making it difficult for them to have a good capacity for self‐compassion. We suggest that direct experience and thorough observation of experiences can help individuals develop higher cognitive flexibility. This allows individuals to have more solutions in difficult situations, including self‐compassion, an adaptive way of being kind to oneself, instead of harsh self‐criticism (Marshall and Brockman 2016). Cognitive flexibility also helps individuals realize that everyone encounters difficulties, rather than believing that they are the only ones suffering misfortune, which means that their capacity for self‐compassion is enhanced. Based on this:

Hypothesis 4 *Cognitive flexibility and self‐compassion play a chain‐mediating role in the relationship between mindfulness and shame*.

### 1.3 The Predictive Effect of Mindfulness on Shame

As mentioned earlier, there have been several cross‐sectional studies validating the negative correlation between mindfulness and shame (Sedighimornani 2019; Brem et al. 2017; Woods and Proeve 2014). There have also been experimental studies validating the impact of mindfulness on shame in clinical interventions (Galhardo, Cunha, and Pinto‐Gouveia 2013; Goldsmith et al. 2014; Proeve, Anton, and Kenny 2018; Westerman, McCann, and Sparkes 2020; Oren‐Schwartz et al. 2022). All of these studies reflect that mindfulness is one of the factors that influence the level of shame. However, these studies mainly focused on Western countries where English is the native language, limiting their applicability in the Chinese region. Qian, Liu, and Zhu (2001) found that Chinese college students experienced shame without a strong sense of insignificance, but still accompanied by painful emotional and physical reactions. Otherwise, most of the subjects were special clinical patients, limiting their applicability to the general population in everyday situations. Some researchers have begun to focus on the influence of mindfulness on shame in Chinese adolescent populations (Zhang et al. 2023). Considering the characteristics of the subject group and the definition of shame in different cultures, we will test the longitudinal relations in the Chinese general adult population in daily life.

Hypothesis 5 *Mindfulness remains an influential cause of shame in the Chinese general adult population*.

We conduct two studies to investigate the effects of mindfulness on shame and to elucidate the mechanisms through which cognitive flexibility and self‐compassion contribute to this relationship. Study 1 tests the relationship between mindfulness and shame through a cross‐sectional study, and the chain‐mediated role of cognitive flexibility and self‐compassion in it. Study 2 examines the longitudinal relations between mindfulness and shame through a longitudinal study lasting 8 months. This research aims to explore the predictive effect of mindfulness on shame to provide more representative, stable, and generalizable findings, and offer ideas for finding more targeted means to effectively intervene with shame.

## 2 Study 1

Study 1 tests the relationship between mindfulness and shame through a cross‐sectional study and the mediating role of cognitive flexibility and self‐compassion.

### 2.1 Method

#### 2.1.1 Participants and Procedure

This study adopts convenient sampling method to conduct online questionnaire survey among adults in Beijing, Chengdu, and other places. After eliminating invalid questionnaires such as incorrect answers and regular answers, the valid sample was 481 people. The age of the subjects ranged from 18 to 45 (26.14 ± 6.46) years. There were 229 males (47.6%) and 252 females (52.4%). Among all the participants, 27 (5.6%) had high school or lower education, 337 people (70.1%) obtained a degree from a college or undergraduate, 117 people (24.3%) obtained master degree or above.

The data were collected through a data collection website (Wenjuanxing, www.wjx.cn). After completing and passing the audit, the participants were given red envelopes to express their gratitude. Based on Monte Carlo power analysis simulation for estimating the sample size required for a mediation effect model (Schoemann, Boulton, and Short 2017). The target power was set to 0.8, and the total sample size needed for a chained mediation model was 351. To ensure the validity of the collected data, a total of 534 questionnaires were gathered. After excluding invalid responses such as those with incorrect answers to detection questions and patterned responses, a final valid sample of 481 questionnaires was obtained. All participants voluntarily took part in the study and, in accordance with the requirements of the Ethics Committee of the Institute of Psychology, Chinese Academy of Sciences; all participants signed an informed consent form.

### 2.2 Measures

#### 2.2.1 Short‐Form Five Facet Mindfulness Questionnaire (FFMQ‐SF)

The FFMQ‐SF developed by Baer et al. (2006) and revised by Zhong et al. (2016) was used in this study. The scale consists of five dimensions, which are observing, describing, acting with awareness, not judging, and not reacting. There are a total of 20 question items, of which eight are reverse scored. A 5‐point Likert scale was used, ranging from 1 “*strongly disagree*” to 5 “*strongly agree*”. The total score of each dimension was used as the mindfulness score in this study, with higher scores representing higher levels of mindfulness among the subjects. An example is “I am good at describing my emotions verbally.” The Cronbach's alpha coefficient for this scale in this study was 0.73; McDonald's ω coefficient was 0.70. The Cronbach's alpha coefficients for the observing, describing, acting with awareness, non‐judging and non‐involving were 0.75, 0.78, 0.83, 0.59, 0.43; the McDonald's ω coefficients were 0.75, 0.78, 0.83, 0.60, 0.48.

#### 2.2.2 Cognitive Flexibility Inventory (CFI)

The CFI developed by Dennis and Vander Wal (2010) and revised by Wang et al. (2016) was used. This section contains 20 items and is scored on a 5‐point Likert scale, with higher scores indicating higher levels of cognitive flexibility. The Cronbach's alpha coefficient for this scale in this study was 0.90; the McDonald's ω coefficient was 0.90.

#### 2.2.3 Self‐Compassion Scale (SCS)

The Chinese version of the SCS developed by Neff (2003b) and revised by Chen, Yan, and Zhou (2011) was used. The section contains 26 items and is scored on a 5‐point Likert scale, with higher scores indicating higher levels of cognitive flexibility. The Cronbach's α coefficient for this scale in this study was 0.87; the McDonald's ω coefficient was 0.87.

#### 2.2.4 Test of Self‐Conscious Affect–3 (TOSCA‐3)

The TOSCA‐3, developed by Tangney and Dearing (2002) was used to assess shame tendency (Ma et al. 2019). The scale contains a total of 16 scenarios in which participants imagined various situations they might encounter in their daily lives and responded to the likelihood of responding in the manner described. A 5‐point Likert scale was used, ranging from 1 “*not at all likely*” to 5 “*very likely*” Only Shame scores were tested in this study, with higher scores representing higher levels of shame in the subjects. The Cronbach's alpha coefficients for the Shame subscale in this study were 0.84; the McDonald's ω coefficient was 0.85, respectively.

### 2.3 Statistical Analysis

SPSS 26.0 (Pallant 2020) was used in this study for descriptive, reliability and validity analyses of the data. The Harman one‐way test was used to test for common method bias for the data; Pearson correlation analysis was used to explore the correlation between mindfulness and shame in the two surveys; and Mplus8.3 was used to conduct the mediating effect analysis.

### 2.4 Results

#### 2.4.1 Common Method Bias Test

In the investigation of this study, the use of multiple self‐assessment questionnaires to measure the same group of people easily leads to the problem of common method bias. Therefore, the influence of the self‐assessment method on the research results was minimized through the mixed distribution of positive and negative questions, and the promise made to the subjects during the data collection process that the data obtained would be used only for scientific research. Meanwhile, Harman one‐way test for common method bias (*n* = 481) of Experiment 1 was used. The results showed that there were 18 factors with eigenvalues greater than 1, and the amount of variance explained by the first factor was 17.61%, which was less than the critical criterion of 40%, so the common method bias in this study was not significant (Zhou and Long 2004).

#### 2.4.2 Descriptive Statistics and Correlations

The mean, standard deviation and correlation analyses of the variables are shown in Table 1, which shows that mindfulness was significantly and positively correlated with cognitive flexibility (*r* = 0.62, *p <* 0.001), self‐compassion (*r* = 0.59, *p <* 0.001), and significantly and negatively correlated with shame (*r* = −0.24, *p <* 0.001). Cognitive flexibility was significantly positively correlated with self‐compassion (*r* = 0.63, *p <* 0.001). Shame was significantly negatively correlated with cognitive flexibility (*r* = −0.33, *p <* 0.001), and self‐compassion (*r* = −0.40, *p <* 0.001). In addition, the dimensions of mindfulness of action of awareness (*r* = −0.33, *p <* 0.001) and nonjudgment (*r* = −0.30, *p <* 0.001) were significantly negatively correlated with shame, and all dimensions of mindfulness were significantly positively correlated with cognitive flexibility and self‐compassion.

**TABLE 1:** Intercorrelations among variables and descriptive statistics.

Variables	1	2	3	4	5	6	7	8	9	10	11	12
1. FFMQ	1											
2. Observe	0.59***	1										
3. Describe	0.68***	0.42***	1									
4. Act aware	0.64***	0.04	0.22***	1								
5. Nonjudge	0.34***	−0.18***	−0.08	0.32***	1							
6. Nonreact	0.49***	0.38***	0.30***	0.02	−0.16***	1						
7. CF	0.62***	0.32***	0.44***	0.50***	0.10*	0.31***	1					
8. SC	0.59***	0.17***	0.28***	0.56***	0.26***	0.34***	0.63***	1				
9. SK	0.47***	0.08	0.20***	0.49***	0.29***	0.21***	0.47***	0.88***	1			
10. CH	0.49***	0.17***	0.23***	0.45***	0.19***	0.29***	0.58***	0.85***	0.62***	1		
11. MF	0.58***	0.20***	0.31***	0.51***	0.19***	0.38***	0.60***	0.87***	0.64***	0.64***	1	
12. Shame	−0.24***	0.09	−0.06	−0.33***	−0.30***	−0.03	−0.33***	−0.40***	0.39***	0.38***	0.26***	1
M	65.00	14.38	13.75	12.4	10.62	13.78	70.96	83.84	32.27	26.06	25.51	47.66
SD	8.32	3.11	3.01	3.58	2.8	2.41	10.4	12.44	5.32	4.27	4.71	10.53

Abbreviations: CF, cognitive flexibility; SC, self−compassion; SK, self−kindness; CH, the sense of common humanity; MF, mindfulness.

* *p* < 0.05.

*** *p* < 0.001.

#### 2.4.3 Chained Mediation Effect Test

Mplus8.3 was used to analyze the mediating effect of cognitive flexibility and self‐compassion between mindfulness and shame. The significance of the mediation effect was measured using Bootstrap with 5000 repeated samples for estimation of 95% confidence intervals.

Using mindfulness as the independent variable, shame as the dependent variable, and gender, age and education as the control variables. The results found that mindfulness significantly negatively predicted shame (*β* = −0.28, *p <* 0.001), that is, there was an unmediated direct effect of mindfulness on shame. With gender, age, and education controlled, cognitive flexibility and self‐compassion were used as mediators between mindfulness and shame, where cognitive flexibility points to self‐compassion. The results showed that the model fit was acceptable: *χ* ^2^ = 257.09, *df* = 120, *χ* ^2^/*df* = 2.14, RMSEA = 0.05, CFI = 0.97, TLI = 0.96, SRMR = 0.04. The specific relationships are shown in Table 2. The mediation result is shown in Table 3.

**TABLE 2:** Regression analysis of the Mindfulness model.

Outcome variable	Predictor variable	β	SE	t	*p*
Cognitive flexibility	Mindfulness	0.71	0.04	20.40	< 0.001
Self‐compassion	Mindfulness	0.42	0.07	6.17	< 0.001
	Cognitive flexibility	0.39	0.07	5.77	< 0.001
Shame	Mindfulness	0.11	0.09	1.28	0.200
	Cognitive flexibility	−0.19	0.09	−2.23	0.026
	Self‐compassion	−0.36	0.07	−4.95	< 0.001

*Note:* The direct predictive effect of mindfulness on shame emotions was not significant (*β* = 0.11, *p* > 0.05), and the mediating effects of cognitive flexibility and self−compassion were significant, suggesting full mediation, with a mediation effect value of −0.39. This is shown in Table 4.

**FIGURE 1:**
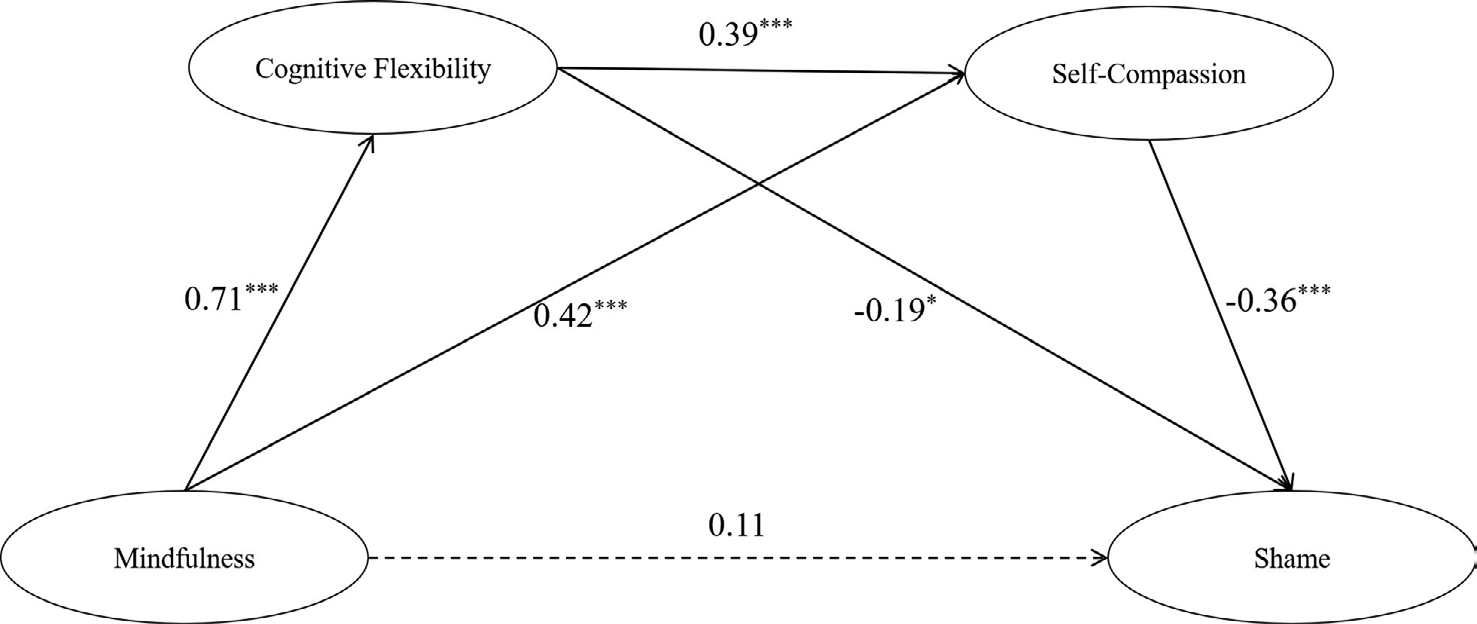
Chain‐mediated model of cognitive flexibility and self‐compassion. *p < 0.05, **p < 0.01, ***p < 0.001.

The mediating effect was generated through 3 mediating paths, with mediating path 1 being mindfulness → cognitive flexibility → shame, with a 95% confidence interval of [−0.26, −0.01] not containing 0, indicating a significant mediating effect of cognitive flexibility, with a mediating effect value of −0.14 and a relative mediating effect of 35.90%. Specifically, mindfulness positively predicted cognitive flexibility (*β* = 0.71, *p <* 0.001), and cognitive flexibility negatively predicted shame (*β* = −0.19, *p <* 0.05). Mediating path 2 was mindfulness → self‐compassion → shame, with a 95% confidence interval of [−0.25, −0.09] not including 0, indicating a significant mediating effect of self‐compassion, with a mediation effect value of −0.15 and a relative mediation effect of 38.46%. Specifically, mindfulness positively predicted self‐compassion (*β* = 0.42, *p <* 0.001), and self‐compassion negatively predicted shame (*β* = −0.36, *p <* 0.001). Mediation path 3 was a chain mediation of mindfulness → cognitive flexibility → self‐compassion → shame, with a 95% confidence interval of [−0.19, −0.06] not including 0, indicating a significant chain mediation of cognitive flexibility and self‐compassion, with a mediation effect value of −0.10 and a relative mediation effect of 25.64%. All three indirect effect paths reached the significant level, and the specific path coefficient results are shown in Figure 1.

### 2.5 Discussion

The present study found that mindfulness could negatively predict shame, and it could also predict shame completely indirectly through cognitive flexibility and self‐compassion and their chained effects.

First, the results of the present study indicated a significant negative correlation between mindfulness and shame, that is, mindfulness is a significant negative predictor of shame, a finding that is consistent with previous research (Woods and Proeve 2014; Brem et al. 2017; Sedighimornani 2019), Hypothesis 1 was testified. In the present study, the two dimensions of mindfulness of acting with awareness and nonjudgment were negatively related to shame. As explained by Shapiro et al. (2006), mindfulness helps individuals allow all experiences to exist in the present moment, and even if these experiential events are not in line with the individual's expectations, an open and friendly attitude can still be maintained in response. This nonjudgmental attitude allows the individual to better focus on what needs to be done in the present moment and encourages the individual to find ways to resolve the dilemma. This process avoids inter‐individual cognitive resources from focusing on concerns and resistances that do not match expectations, and thus prevents the individual from immersing him/herself in denial of his or her own abilities. As a result, individuals do not automate their denial of self because things do not go as expected, and shame levels are alleviated as a result.

Second, this study found that cognitive flexibility mediates the relationship between mindfulness and shame. That is, the higher the level of mindfulness, the higher the cognitive flexibility and the less shame that resulted. This finding confirms the results of previous research on the relationship between cognitive flexibility and shame (Cenkner, Usman, and Zalta 2023; Cenkner, Held, and Zalta 2024), and Hypothesis 2 was supported. Individuals who direct their attention to facing the dilemma they are experiencing in a nonjudgmental manner, rather than focusing exclusively on negative concerns about the self, have the opportunity to develop greater cognitive flexibility. This can help individuals reintegrate cognitive resources and find more adaptive ways to face difficulties. On the one hand, individuals may find more solutions to difficulties to achieve their goals; on the other hand, individuals have the opportunity to explain why the current situation arose from other perspectives, replacing the total rejection of their own abilities, and thus have the potential to reduce shame.

In addition, this study found that self‐compassion mediates the relationship between mindfulness and shame. That is, the higher the level of mindfulness, the higher the level of self‐compassion, and the less shame that resulted. This result is consistent with previous findings on the role of self‐compassion between mindfulness and shame (Sedighimornani 2019; Woods and Proeve 2014), and Hypothesis 3 was testified. Additionally, the three dimensions of self‐compassion are all significantly negatively correlated with the tendency toward shame. This means that in addition to the level of mindfulness being related to the reduction of shame, both the sense of common humanity and self‐kindness are closely associated with the decrease in shame, which is in line with the experimental findings of Ceclan and Nechita (2021). Mindfulness and self‐compassion are closely related and different in that while both emphasize a nonjudgmental attitude toward present‐moment experiences, mindfulness places more emphasis on allowing all experiences to exist, whereas self‐compassion is more directed toward painful experiences (Sun et al. 2021). Individuals may experience suffering when their expectations are not aligned with the present situation. Self‐compassion assists individuals in becoming more aware of their needs in the present moment and in providing themselves with the necessary warmth and support. This support encompasses being kind to oneself and connecting with others. As Neff (2003a) pointed out, mindfulness serves as the foundation of self‐compassion. Mindfulness guides the individual to experience the present moment in an open, nonjudgmental manner and allows such situations to exist. Such allowing does not mean doing nothing, but helps individuals become further aware of their current needs and express kindness to themselves by meeting those needs. On the one hand, the self‐compassionate part of self‐compassion helps the individuals understand and care about themselves instead of being self‐critical; on the other hand, the aspect of common humanity helps individuals shift their focus from beliefs like “Why is it always me who fails” and instead seek connection with others, thereby reducing feelings of loneliness. This can aid individuals in avoiding harsh self‐criticism and blame, and counteract potential feelings of shame that arise from a sense of disconnection and the feeling of being ostracized for making mistakes. The mindfulness component of self‐compassion guides individuals to clearly perceive the current situation without overly identifying with these painful experiences of failure. As a result, they do not engage in comprehensive and uncontrollable negative self‐evaluations, thus reducing shame.

Finally, this study found that mindfulness influences shame through the chain mediation of cognitive flexibility and self‐compassion, and Hypothesis 4 was tested. The ability to have high cognitive flexibility helps individuals realize that there are multiple explanations for what creates difficulties in the moment (Dennis and Vander Wal 2010). On the one hand, this allows individuals to have more perspectives on suffering and to more readily realize that they and others may experience such difficulties, and therefore stop focusing on the parts of themselves that they are not doing well; on the other hand, cognitive flexibility also allows individuals to have the ability to apply more ways of coping with difficulties, including treating themselves in a friendly manner, replacing harshness on the self in lieu of criticizing the self. In addition to reducing self‐criticism, individuals with high cognitive flexibility can also alleviate shame by increasing their sense of common humanity. Since individuals who feel ashamed often cope with the current situation through avoidance, withdrawal, and other means, this implies a greater likelihood of experiencing loneliness (Vliet 2008). When individuals wish to treat themselves kindly through connecting with others, they first need cognitive resources to realize that there are people they can connect with, or to recognize that the difficulties they are experiencing are sufferings that everyone might encounter, and not something they are facing alone.

Overall, the th mediating paths in this study hold true. This suggests that whether it is by changing cognition to cope with difficulties or by changing attitudes toward the self, it can be a good way to help individuals avoid focusing on negative evaluations of the self to produce shame.

## 3 Study 2

Study 2 suggested a longitudinal association between mindfulness and shame at two time‐periods over an 8‐month period.

### 3.1 Method

#### 3.1.1 Participants and Procedure

This study used convenience sampling to conduct a longitudinal follow‐up survey of adults in Beijing and Chengdu over an 8‐month period. A total of 2 questionnaire tests were conducted, with an interval of 8 months, with subjects completing the questionnaire at the first time point (T1) and completing the same questionnaire at the second time point (T2). After excluding incorrect answers to probe questions, regular responses, or mismatched data, the final valid follow‐up sample was 128. The age range of the subjects was 19~44 (29.98 ± 6.84) years old, with 48 (37.5%) males and 80 (62.5%) females; education level: 4 (3.2%) in high school and below, 93 (72.6%) in college and bachelor's degree, and 31 (24.2%) in master's degree and above. All participants voluntarily took part in the study and, in accordance with the requirements of the Ethics Committee of the Institute of Psychology, Chinese Academy of Sciences, all participants signed an informed consent form.

In a recent cross‐lagged study on shame, the effective sample size was 96 after a 6‐month interval, and it decreased to 85 after a 12‐month interval (Nechita and David 2023). Based on these data, the target sample size for this study was set at 120. The first survey time (T1) was March 2023, a total of 157 questionnaires were collected, excluding the probing questions answered incorrectly and the regular answering questionnaires, 152 valid questionnaires were retained, and the validity rate was 96.81%; the second survey time (T2) was November 2023, a total of 136 questionnaires were collected, excluding the probing questions answered incorrectly and the regular answering questionnaires, 132 valid questionnaires were retained, and the validity rate was 97.06%. The final valid sample was the 128 data that all completed the two tests.

### 3.2 Measures

#### 3.2.1 Short‐Form Five Facet Mindfulness Questionnaire (FFMQ‐SF)

As in Study 1, the Chinese version of FFMQ‐SF was used to measure participants' level of mindfulness. The Cronbach's α coefficient for this scale in study 2 was 0.79 at T1 and 0.80 at T2, and the McDonald's ω coefficient was 0.79 at T1 and 0.73 at T2.

#### 3.2.2 Test of Self‐Conscious Affect–3 (TOSCA‐3)

As in Study 1, the TOSCA‐3 was used to assess shame tendency (Ma et al. 2019). In study 2, the Cronbach's α coefficient for the shame subscale was 0.81 at T1 and 0.81 at T2, and the McDonald's ω coefficient was 0.81 at T1 and 0.81 at T2.

### 3.3 Statistical Analysis

SPSS 26.0 and Mplus 8.3 software were used to analyze the data in this study. The Harman one‐way test was used for the common method bias test for the data. Pearson correlation analysis was used to explore the correlation between mindfulness and shame in the two measures. Mplus8.3 was used to fit a cross‐lagged model of mindfulness and shame.

### 3.4 Results

#### 3.4.1 Common Method Bias Test

An exploratory factor analysis was conducted using the Harman one‐way test for all question items in Study 2 to test for common method bias (*n* = 128) in the two surveys. The results showed that at the first measurement, there were 27 factors with eigenvalues greater than 1, with the first factor explaining 18.25% of the variance. At the second measurement, there were 26 factors with eigenvalues greater than 1, of which the first factor explained 19.26% of the variance, which is less than the critical criterion of 40%. Therefore, there is no common method bias in this study (Zhou and Long 2004).

#### 3.4.2 Descriptive Statistics and Correlation Analysis

Descriptive statistics for the two time points are shown in Table 4, which includes means and standard deviations. The results showed that T1 mindfulness showed a significant positive correlation with T2 mindfulness (*r* = 0.73, *p <* 0.001), and T1 shame with T2 shame (*r* = 0.72, *p <* 0.001), that is, a stable correlation between the variables; T1 mindfulness showed a significant negative correlation with T1 shame (*r* = −0.21, *p <* 0.05), and T2 mindfulness with T2 shame showed a significant negative correlation (*r* = −0.34, *p <* 0.001), that is, synchronicity correlation between variables. The basic assumptions of the cross‐lagged design were satisfied because the stability and synchronicity correlation between the variables were satisfied at the same time.

**TABLE 3:** Cross−lagged model path coefficients.

Influence path	Standardized coefficients	SE	*p*
Mindfulness T1 → mindfulness T2	0.71	0.05	< 0.001
Shame T1 → shame T2	0.65	0.05	< 0.001
Mindfulness T1 → shame T2	−0.18	0.06	0.002
Shame T1→ mindfulness T2	−0.04	0.07	0.513

**TABLE 4:** Mediating effect analysis of the mindfulness model.

Influence path	Indirect effect	BootSE	95% confidence interval	Relative mediating effect
Total	−0.39	0.07	[−0.52, −0.26]	—
Mindfulness → CF → shame	−0.14	0.06	[−0.26, −0.01]	35.90%
Mindfulness → SC → shame	−0.15	0.04	[−0.25, −0.09]	38.46%
Mindfulness → CF → SC → shame	−0.10	0.03	[−0.19, −0.06]	25.64%

Abbreviations: CF, cognitive flexibility; SC, self−compassion.

#### 3.4.3 Cross‐Lag Analysis of Mindfulness and Shame

**FIGURE 2:**
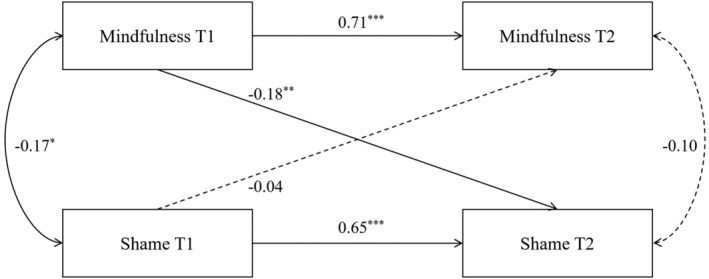
The cross‐lagged model of mindfulness and shame. *p < 0.05, **p < 0.01, ***p < 0.001.

On the basis of correlation analysis, the cross‐lagged analysis was used to explore the interactive predictive relationship between mindfulness and shame. The cross‐lagged structural equation model with fixed effects was constructed using Mplus, and the age and gender of the subjects in the T1 period were introduced into the model as control variables, as shown in Figure 2.

After controlling for T1 shame, T1 mindfulness negatively predicted T2 shame (*β* = −0.18, *p <* 0.01); after controlling for T1 mindfulness, T1 shame did not predict T2 mindfulness (*β* = −0.04, *p >* 0.05). This result can be taken as preliminary evidence that there is a longitudinal association between mindfulness and shame, that is, mindfulness predicts shame, but shame does not predict mindfulness. The specific coefficients are shown in Table 5.

**TABLE 5:** Results of descriptive statistics and correlation analysis for each variable.

	M	SD	1	2	3
1. Mindfulness T1	64.80	8.97	—		
2. Shame T1	44.64	9.64	−0.21*	—	
3. Mindfulness T2	65.53	8.50	0.73***	−0.23**	—
4. Shame T2	46.86	8.89	−0.33***	0.72***	−0.34***

* *p* < 0.05.

** *p* < 0.01.

*** *p* < 0.001.

### 3.5 Discussion

In this study, it was found that adults' mindfulness level predicted their shame level 8 months later, and Hypothesis 5 was verified. This result supports a potential strategy to reduce shame by enhancing mindfulness. While shame‐prone individuals often attribute failures to their own inability, mindfulness implies allocating more attention to the awareness of the here and now experience and emphasizes that such awareness is manifested in a nonjudgmental attitude. Adopting this kind of awareness allows individuals to have a clearer perception of their inner emotional thoughts (Xu, Wang, and Liu 2015) and a clearer understanding of their inner workings. It can help individuals treat negative evaluations of the self as merely thoughts brought about by inner work, rather than facts (Liu et al. 2015). Thus, individuals with high levels of mindfulness can more easily shift attention from negative evaluations of the self to the experience of the present moment, see thoughts as only thoughts, and reduce over criticism of the self, thereby reducing shame levels. On the other hand, shame‐prone individuals are more susceptible to external evaluations and pay too much attention to what others say about them (Gilbert 1998). Mindfulness also helps individuals clearly appreciate their concern about others' evaluations and prevent getting involved in the whirlwind of information tied to others.

Second, an individual's level of shame did not predict the level of mindfulness 8 months later. This suggests that mindfulness is not only an awareness that encompasses self‐awareness (Kabat‐Zinn 2003), but also a series of mental processes related to self‐regulation and metacognition (Bishop et al. 2004), which are relatively stable to some extent (Xu, Wang, and Liu 2015). Moreover, mindfulness emphasizes not recalling the past nor thinking about the future, but simply experiencing the present moment as it is, and taking these experiences to a new examination of one's perceptions in a nonjudgmental manner (Shapiro et al. 2006). Thus, shame experiences do not significantly predict future levels of mindfulness in individuals. If mindfulness is compared to an ocean, shame that focuses on one's own negative evaluations is like a wave in the ocean and does not have any effect on the ocean.

In this study, a longitudinal survey was conducted in a Chinese adult population, and the results of cross‐lagged analyses provided evident support for the negative predictive effect of individual mindfulness levels on shame. That is, higher levels of mindfulness predicted lower levels of shame. Therefore, individuals can try to enhance their mindfulness level to alleviate shame by means of mindfulness training. This has theoretical and practical implications for individuals to maintain mental health and improve quality of life.

## 4 General Conclusion

The present study explored the mechanism of mindfulness in alleviating shame through a cross‐sectional approach and established the predictive role of mindfulness on shame via a longitudinal research design. The findings of the present study extend our understanding of the intricate relationships between mindfulness, cognitive flexibility, self‐compassion, and shame, offering both theoretical and practical implications.

From a theoretical perspective, this research supports the growing body of evidence that mindfulness not only plays a direct role in mitigating shame but also does so indirectly through enhancing cognitive flexibility and self‐compassion. This reflects the broader theoretical framework suggesting that mindfulness can promote adaptive emotional regulation by enabling individuals to reinterpret and detach from negative emotional experiences, such as shame. The findings also support existing theories suggesting that mindfulness facilitates adaptive thinking patterns, allowing individuals to reframe negative experiences more effectively (Keng, Smoski, and Robins 2011). By improving cognitive flexibility, individuals become better equipped to shift their perspectives and reduce rigid thinking associated with shame (Cenkner, Usman, and Zalta 2023; Martin and Rubin 1995). Additionally, the mediating role of self‐compassion aligns with Neff's (2003a) framework, which posits that self‐compassion mitigates self‐critical emotions by promoting kindness and understanding toward oneself.

Practically, these insights suggest that mindfulness‐based interventions (MBIs) can be strategically designed to target shame by incorporating components that enhance cognitive flexibility and self‐compassion. First, the findings emphasize the potential of MBIs to specifically target and reduce shame in non‐clinical populations. Given the negative consequences of shame on mental health, including its association with anxiety, depression, and other psychological conditions, these interventions can be valuable tools for both therapeutic and preventive purposes. Additionally, by highlighting the mediating roles of cognitive flexibility and self‐compassion, this research suggests that enhancing these qualities could be integral components of mindfulness‐based therapeutic approaches. For example, MBIs could be tailored to explicitly focus on building cognitive flexibility—helping individuals to shift away from rigid patterns of thinking that reinforce shame (Beshai, Prentice, and Huang 2018)—and fostering self‐compassion, encouraging more kind and supportive internal dialogues (Mohajeri, Alfooneh, and Imani 2023; Daneshvar, Basharpoor, and Shafiei 2022).

Moreover, the longitudinal aspect of the study underscores the sustained benefits of mindfulness in reducing shame over time, which is crucial for developing long‐lasting interventions. Practitioners can be confident that incorporating mindfulness into therapeutic settings not only addresses immediate emotional concerns but also fosters long‐term resilience against shame. By implementing mindfulness‐based strategies, individuals can develop tools to better manage shame in their everyday lives, ultimately promoting better emotional and mental health in the long run.

There are some shortcomings in this research. First of all, this study used convenience sampling to select subjects and had a limited number of subjects, which may affect the effect size. Second, this study used the TOSCA‐3 with a Chinese revision to test shame. In the future, more types of scales can be administered to validate the results of different types of shame. Third, this study only demonstrated the predictive effect of mindfulness on shame in the longitudinal dimension. The effects of mindfulness dimensions, cognitive flexibility, and self‐compassion in the longitudinal relationship have not yet been investigated, and their longitudinal mechanisms of shame can be further analyzed in the future studies.

In summary, the present study not only demonstrated the relationship between mindfulness and shame in Chinese adults, but also elucidated the underlying mechanism, that is, chain‐mediated influence through cognitive flexibility and self‐compassion. Future research on the role of cognitive flexibility, self‐compassion, and mindfulness interventions in reducing shame warrants further exploration in order to develop more reliable and targeted interventions to alleviate shame.

## Ethics Statement

The studies were conducted in accordance with the Declaration of Helsinki, and approved by the Institutional Review Board (or Ethics Committee) of The Institute of Psychology, Chinese Academy of Sciences.

## Consent

Informed consent was obtained from all participants involved in the study.

## Conflicts of Interest

The authors declare no conflicts of interest.

## Data Availability

The datasets generated and/or analyzed during the current study are available from the corresponding author upon reasonable request.
